# Effective mortality thresholds for reporting suspicion of highly pathogenic avian influenza in mule ducks

**DOI:** 10.1186/s13567-025-01525-9

**Published:** 2025-04-19

**Authors:** Sébastien Lambert, Caroline Godard, Timothée Vergne

**Affiliations:** IHAP, Université de Toulouse, INRAE, ENVT, Toulouse, France

**Keywords:** Mule ducks, avian influenza, mortality, surveillance, early detection, poultry

## Abstract

Effective mortality thresholds are critical for timely reporting and management of highly pathogenic avian influenza. Using standard modelling techniques, we evaluated the performance of different mortality thresholds in mule duck flocks. Using an eightfold increase of the mortality for two consecutive days compared to the average mortality the previous week led to a perfect classification of all flocks used for validation (12 affected and 18 non-affected flocks). A fixed daily threshold of 0.25% showed a perfect sensitivity and a good specificity (3 false positives/18). Our results fill a knowledge gap and can inform HPAI surveillance policy in non-vaccinated mule ducks.

**Supplementary Information:**

The online version contains supplementary material available at 10.1186/s13567-025-01525-9.

## Introduction, methods, and results

In animal health, the detection and reporting by farmers of abnormal clinical signs or mortality events is at the forefront of passive surveillance [1], especially for high morbidity and high mortality infectious diseases such as highly pathogenic avian influenza (HPAI). In the absence of vaccination, sensitive and timely reporting, quickly followed by depopulation of infected flocks, is essential to limit the spread of such deadly pathogens [2, 3]. Between 2016 and 2024, recurrent epizootics of HPAI viruses have spread widely in Europe and caused thousands of outbreaks in poultry farms [4], calling for effective surveillance strategies [5]. Using appropriate thresholds to report sudden changes in daily mortality has been shown to be sensitive and timely to detect HPAI circulation in poultry flocks [6–10].

Various mortality thresholds have been defined in Europe for mandatory reporting of suspicions by farmers. In 2000, because of the HPAI H7N1 epidemic in Italy, the Dutch veterinary authorities set a daily mortality threshold of 0.5% for one day. It was replaced in 2003 during the Dutch HPAI H7N7 epidemic by a weekly threshold of 3% [11], also used in the European legislation [12]. A retrospective analysis of the 2003 Dutch epidemic showed that this threshold was reached two or three days after the start of increased mortality, and recommended instead thresholds between 0.25% and 1% per day for two consecutive days in chicken layers and turkeys [11]. These thresholds were implemented in the Dutch legislation in 2005 [7, 8]. In France, similar thresholds were used, with the additional criteria that the mortality in the second day is at least twice that of the first day [13]. Mortality thresholds of 2% or 4% for one day were also used for palmipeds and galliforms, respectively [13]. In addition, an increase in the daily mortality of more than 3 times the normal mortality rate of the flock was used [14], following the European Commission decision 2006/437/EC [15].

Recent studies found efficient fixed mortality thresholds of 0.08% or 0.13% for chicken layer flocks kept indoors or outdoors, respectively [7], 0.3% for Pekin duck flocks [9], and 0.17% for chicken broiler flocks [10]. A 2.9 times higher mortality than the average mortality of the previous week was also suggested as an effective threshold [7, 8]. To our knowledge, similar analyses have not yet been performed for the detection of HPAI outbreaks in mule duck flocks. Mule ducks are cross-bred between Pekin ducks and Muscovy ducks, and are commonly used for fat duck production in France, Bulgaria and Hungary. Their production differs from that of typical Pekin ducks: during the first production phase (“breeding phase”), mule ducks are raised outside from 3–4 weeks of age to 11–14 weeks of age, before they are moved to other farms for the second production phase (“fattening phase”) which lasts 10 to 14 days [16]. To fill this knowledge gap, we used a similar method as [7, 9, 10] to find efficient mortality thresholds to detect HPAI outbreaks in French mule duck flocks during the first production phase.

First, retrospective daily mortality records from 18 non-HPAI-infected flocks were provided by the poultry industry. These daily mortality incidence, which are manually written by farmers on production calendars, were transformed into electronic spreadsheets to enable analyses with the statistical software R version 4.3.3 [17]. To estimate the expected daily baseline mortality in French mule duck flocks, we fitted a generalised linear mixed model (GLMM) where the daily number of dead ducks was the response variable, the natural logarithm of the daily population size of the flock was the offset, and the age of ducks in days was the explanatory variable. To account for deviations in linearity during the production cycle, natural cubic splines were used on the variable age [18] (Additional file 1). The flock identifier was used as the grouping variable (random effect). We assessed a Poisson and a negative binomial distribution, and we selected the latter model as it produced a better fit and accounted for the overdispersion observed with a Poisson distribution (Additional file 1).

**Figure 1:**
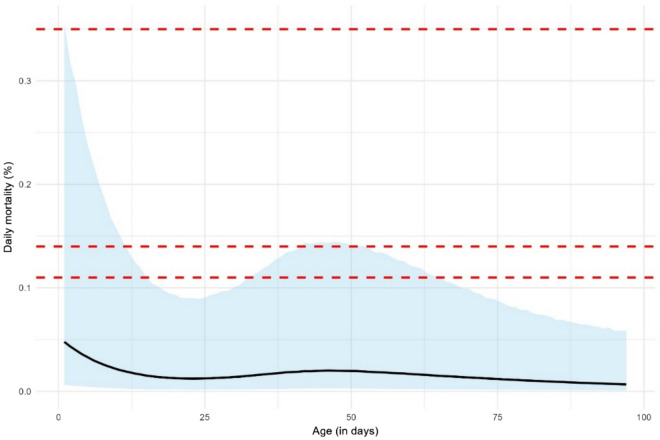
**Expected daily mortality in French mule duck flocks according to the negative binomial generalised linear mixed model (line: mean; blue area: 95% prediction interval)**. Thresholds (red horizontal dashed lines) were defined based on the upper limit of the 95% prediction interval: 0.11% (median), 0.14% (third quartile) and 0.35% (maximum).

Overall, the expected mortality decreased from 0.05% (95% prediction interval—PI: 0.006–0.35%) on the first day, to 0.012% (95% PI: 0.002–0.09%) around three weeks of age (Figure 1). It then increased up to 0.02% (95% PI: 0.003–0.15%) during the sixth week, before decreasing again with a minimum of 0.007% (95% PI: 0.001–0.06%) at the end of the production cycle. From the expected daily baseline mortality, we defined fixed daily mortality thresholds to assess, considering that mortality in non-HPAI infected flocks should be below the upper limit of the 95% PI [7, 9, 10]. As the upper limit of the 95% PI is time-dependent, three thresholds were defined: 0.11% (median), 0.14% (third quartile) and 0.35% (maximum—Figure 1). In addition, we also evaluated the performance of the 0.25% and 2% thresholds defined in the French legislation [13]. These thresholds were tested for one or two consecutive days [7, 9], as well as for two consecutive days with the additional criteria that the mortality in the second day is at least twice that of the first day, to match the French legislation [13].

Using retrospective daily mortality records from 12 HPAI-infected flocks from the 2016–2017 and 2020–2021 epidemics (clade 2.3.4.4b subtype H5N8), we evaluated the sensitivity and the timeliness of the thresholds. When evaluated over a single day, perfect sensitivity was observed for all thresholds except the 2% threshold (Table 1). Detection happened up to 3 days earlier (median: 1 day) with a 0.11% threshold and up to 3 days later (median: 1 day) with a 2% threshold compared to the date of detection with a 0.25% threshold (Additional file 1). Sensitivity and timeliness both deteriorated when evaluating the thresholds for two consecutive days (Table 1 and Additional file 1). The specificity and the number of false alarms per 100 days of production were evaluated using the mortality records from the 18 non-HPAI-infected flocks. In contrast to sensitivity, specificity increased with increasing mortality thresholds when evaluated over a single day, with perfect specificity for the 2% threshold (Table 1). The number of false alarms (50% of which happened during the first three weeks of age) decreased to a mean of 2.3 per 100 days (min: 0–max: 12.3) for the 0.11% threshold to no false alarms for the 2% threshold (Additional file 1). Specificity improved and the number of false alarms decreased when evaluating the thresholds for two consecutive days, with perfect specificity for all thresholds when mortality doubled on the second day (Table 1 and Additional file 1).

**Table 1:** **Performance of mortality thresholds applied to report suspicion of highly pathogenic avian influenza in mule duck flocks.**

Thresholds	Sensitivity			Specificity		
	1 day	2 days	2 days D2: $\ge$2 $\times$ D1	1 day	2 days	2 days D2: $\ge$2 $\times$ D1
Mortality $\ge$ 0.11%	12/12	12/12	11/12	6/18	14/18	18/18
Mortality $\ge$ 0.14%	12/12	12/12	11/12	9/18	15/18	18/18
Mortality $\ge$ 0.25%	12/12	12/12	10/12	15/18	16/18	18/18
Mortality $\ge$ 0.35%	12/12	10/12	9/12	15/18	17/18	18/18
Mortality $\ge$ 2%	8/12	–	–	18/18	–	–
Ratio $\ge$ 8.0	12/12	12/12	–	4/18	17/18	–
Ratio $\ge$ 10.7	12/12	12/12	–	5/18	17/18	–
Ratio $\ge$ 17.2	12/12	11/12	–	13/18	17/18	–
Ratio $\ge$ 34.4	12/12	9/12	–	16/18	18/18	–
Moving average $\ge$ 7.4	12/12	11/12	–	6/18	18/18	–
Moving average $\ge$ 8.6	12/12	8/12	–	6/18	18/18	–
Moving average $\ge$ 10.8	12/12	8/12	–	8/18	18/18	–
Moving average $\ge$ 14.1	12/12	7/12	–	12/18	18/18	–

An alarm was raised on the day a threshold was passed or after two consecutive days, with or without the additional criteria that the mortality in the second day is at least twice that of the first day.

D1: mortality at day 1; D2: mortality at day 2; -: not evaluated.

A similar approach was used to evaluate the mortality ratio, which compares the mortality on a given day to the average mortality of the previous week from the same flock (see Additional file 1 and [7] for more details). Therefore, this ratio has the additional benefit of being flock-specific. As before, three ratio thresholds were defined based on the 95% PI of a GLMM: 8.0, 10.7, 17.2. Based on the performance of these thresholds, we also evaluated the 34.4 threshold (twice the maximum value). When evaluated over a single day, perfect sensitivity was observed for all ratio thresholds, and specificity improved with increasing threshold values (Table 1). When evaluated over two consecutive days (as originally suggested by [7]), perfect specificity and sensitivity was observed for ratio thresholds of 8.0 and 10.7 (Table 1).

We also evaluated a seven-day moving-average [6], but this method did not provide a good balance between sensitivity and specificity (Table 1 and Additional file 1).

## Discussion

Using daily mortality records from mule duck flocks infected or not by HPAI viruses, we evaluated the performance of mortality thresholds following existing methods [7, 9, 10]. Based on our results (Table 1), we recommend using either a fixed mortality threshold of 0.25% for one or two consecutive days, or a mortality ratio of 8.0 for two consecutive days.

Our fixed threshold value of 0.25% is consistent with the 0.30% value previously found for Pekin ducks [9]. The 0.25% value already appeared in the French regulation, but for a suspicion to be raised, mortality had to be above this threshold for two consecutive days and mortality on the second day had to be at least twice that of the first day [13]. However, in our results, the sensitivity for this criterion was not optimal (Table 1). Therefore, we recommend instead to use the 0.25% value over a single day or over two days.

The ratio threshold of 8.0 was higher than the 2.9 value found for layer chickens [7]. A preliminary analysis in Pekin ducks suggested that the same 2.9 value could also be effective for this type of production but needed further evaluation [8]. Here, the 8.0 mortality ratio was highly effective in mule duck flocks with perfect sensitivity (*n* = 12) and specificity (*n* = 18). Therefore, it could represent a good complement or alternative to fixed mortality thresholds, while remaining easy enough to be translated into regulatory texts and for farmers to implement on a daily basis [7].

Other types of flock-specific triggers have been used in the past, e.g., relying on moving averages [6, 7, 9]. In this study, we evaluated a seven-day moving average which did not perform as well as the fixed thresholds or the mortality ratio.

Finally, our work showed time-varying mortality in non-HPAI-infected flocks (Fig. 1). We observed a higher mortality during the first days of life, as in broiler chickens [19], and a second peak around the middle of the production cycle. Determining the factors associated with increased mortality in mule duck flocks could represent a future area of research, to improve production and welfare [19].

Our study suffers from a number of limitations. First, our sample size remains limited, with only 12 HPAI-infected flocks and 18 non-infected flocks. Ideally, our results should therefore be confirmed using a larger sample size. Second, our HPAI-infected flocks all date back to the H5N8 2016–2017 and 2020–2021 epidemics. Therefore, our results may not reflect the behaviour of more recent epidemics, especially as the dominant subtype changed from H5N8 to H5N1 since 2022 [3]. Using data from more recent epidemics, when available, is therefore recommended to confirm or update the threshold values that were found in our study. Finally, our mortality thresholds were based exclusively on mule duck flocks that were not vaccinated against HPAI. Therefore, these thresholds cannot be translated for surveillance in vaccinated mule duck flocks, which currently limit their use in the French context where vaccination is being implemented since 2023 [20]. In vaccinated flocks, this mortality-based passive surveillance strategies should be replaced by other surveillance methods such as systematic sampling and testing of dead ducks every week [20]. Nonetheless, our work should remain beneficial for current or future contexts where vaccination is not implemented, in France or other countries such as Hungary.

## Supplementary Information

**Additional file 1. Additional details on the analyses and the code used, with supplementary results and figures. **

## Data Availability

All the scripts and data that were used for the analyses are available at 10.57745/3ZFSEE.
