# Supplementary material for: Effective mortality thresholds for reporting suspicion of highly pathogenic avian influenza in mule ducks
Source: Vet Res. 2025 Apr 19;56:85. doi: 10.1186/s13567-025-01525-9 (PMC12008835; doi:10.1186/s13567-025-01525-9)
Supplement: Supplementary file 1 — Additional file 1. Additional details on the analyses and the code used, with supplementary results and figures. [file 13567_2025_1525_MOESM1_ESM.pdf]

Additional file: Effective mortality thresholds for reporting suspicion of highly pathogenic avian influenza in mule ducks

Sébastien Lambert, Caroline Godard, Timothée Vergne

Table of content

1 Data **2**

1.1 Healthy flocks 2

1.2 HPAI-infected flocks 2

2 Fixed mortality thresholds **3**

2.1 Expected daily baseline mortality 3

2.2 Mortality above a given threshold for a single day 6

2.3 Mortality above a given threshold for two consecutive days 12

2.4 Mortality that doubles after being above a given threshold 14

3 Mortality ratio **17**

3.1 Expected baseline mortality mortality ratio 17

3.2 Mortality ratio above a given threshold for a single day 20

3.3 Mortality ratio above a given threshold for two consecutive days 21

4 Seven-day moving-average **26**

4.1 Thresholds for the seven-day moving-average trigger 26

4.2 Daily mortality above the moving-average for a single day 28

4.3 Daily mortality above the moving-average for two days 30

1 Data

1.1 Healthy flocks

Daily mortality data recorded by farmers were provided by the poultry industry for 18 healthy (i.e., non-HPAI-infected) French mule duck flocks raised in 2021 and 2022.

```
flocks <- read.csv(
  file = "data/flock_characteristics.csv",
  colClasses = c("factor", "numeric", "logical")
)
dat_healthy <- read.csv(
  file = "data/mortality_healthy_flocks.csv",
  colClasses = c("factor", "numeric", "numeric")
)
```

The characteristics of the flocks are summarised below:

```
summary(flocks$size[flocks$infected == FALSE])
```

```
##    Min. 1st Qu.  Median    Mean 3rd Qu.    Max.
##    2040    5962    7320    7491    8798   19382
```

```
summary(dat_healthy, digits = 2)
```

```
##        id            age        mortality
##  FRNI_03: 97   Min.   : 1   Min.   :  0.0
##  FRNI_15: 85   1st Qu.:20   1st Qu.:  0.0
##  FRNI_07: 84   Median :40   Median :  0.0
##  FRNI_08: 84   Mean   :40   Mean   :  1.5
##  FRNI_09: 84   3rd Qu.:60   3rd Qu.:  2.0
##  FRNI_01: 83   Max.   :97   Max.   :175.0
##  (Other):907
```

1.2 HPAI-infected flocks

Daily mortality data recorded by farmers for 12 HPAI-infected flocks were retrieved from the French official veterinary services. These flocks were infected by HPAI H5N8 viruses in 2016-2017 (n=11) and in 2020-2021 (n=1).

```r
dat_infected <- read.csv(
  file = "data/mortality_infected_flocks.csv",
  colClasses = c("factor", "numeric", "numeric")
)
```

The characteristics of the flocks are summarised below:

```r
summary(flocks$size[flocks$infected == TRUE])
```

```
##    Min. 1st Qu.  Median    Mean 3rd Qu.    Max.
##     918    3692    4804    5313    7034   10506
```

```r
summary(dat_infected, digits = 2)
```

```
##         id            age        mortality
##  FRI_12 : 62   Min.   : 1   Min.   :  0
##  FRI_09 : 54   1st Qu.:12   1st Qu.:  0
##  FRI_03 : 53   Median :24   Median :  0
##  FRI_06 : 53   Mean   :25   Mean   : 14
##  FRI_08 : 53   3rd Qu.:37   3rd Qu.:  3
##  FRI_05 : 50   Max.   :62   Max.   :720
##  (Other):243
```

2 Fixed mortality thresholds

2.1 Expected daily baseline mortality

To estimate the expected daily baseline mortality in French mule duck flocks, we fitted a generalised linear mixed model (GLMM) where the daily number of dead ducks was the response variable, the age of ducks (in days) was the explanatory variable, and the flock identifier was the random effect [7, 9, 10].

We also used the natural logarithm of the daily population size of the flock as the offset, to account for different population sizes among different flocks. For each day, we therefore calculated the number of live ducks before mortality happened:

```r
dat_healthy$popsize <- unlist(sapply(levels(dat_healthy$id), function(j) {
  flocks$size[flocks$id == j] -
    c(0, cumsum(dat_healthy$mortality[dat_healthy$id == j])[
      -sum(dat_healthy$id == j)
    ])
}), use.names = FALSE)
```

Based on this, we also calculated the percentage of death that occurred every day (Figure S1):

```
dat_healthy$propmort <- dat_healthy$mortality / dat_healthy$popsize
```

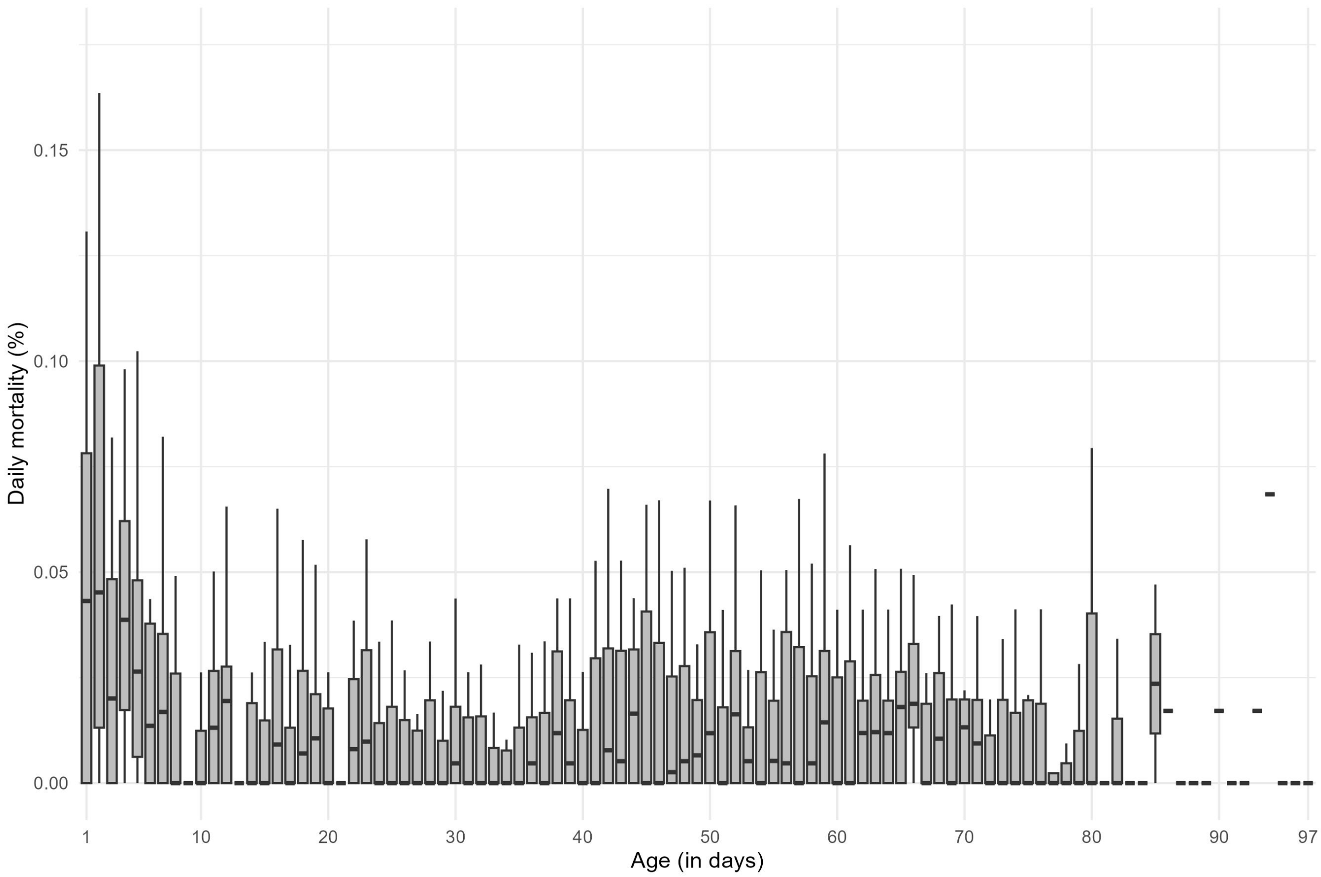

Figure S1: Daily mortality (%) in 18 healthy mule duck flocks.

With the daily population size, we were then able to fit GLMMs to our data. We first assessed a Poisson distribution:

```
mod_pois <-
  lme4::glmer(mortality ~ age + (1 | id) + offset(log(popsize)),
    data = dat_healthy, family = poisson
  )
```

This model had an AIC (Akaike information criterion) of 7111.12, and showed a significant overdispersion (p= 0).

Therefore, we also assessed a negative binomial distribution to account for the observed overdispersion:

```
mod_nb <-
  lme4::glmer.nb(mortality ~ age + (1 | id) + offset(log(popsize)),
    data = dat_healthy
  )
```

This model showed a better fit (AIC=4358.94) and was therefore selected.

To account for deviations in linearity during the production cycle, natural cubic splines were used on the variable age. Following [18], we chose the number of knots by comparing models' AICs, testing up to five knots (i.e., up to six degrees of freedom).

```
aic <- rep(0, 6)
for (df in seq_len(length(aic))) {
  aic[df] <- AIC(
    lme4::glmer.nb(
      mortality ~ splines::ns(age, df) + (1 | id) + offset(log(popsize)),
      data = dat_healthy
    )
  )
}
```

The model with the lowest AIC (4327.72) had 3 knots. Models with four and five knots had a ΔAIC<2, but we selected the most parsimonious model as our final model.

```
final_mod <-
  lme4::glmer.nb(
    mortality ~
      splines::ns(age, which.min(aic)) + (1 | id) + offset(log(popsize)),
    data = dat_healthy
  )
```

We then predicted fitted values and estimated 95% prediction intervals, for each individual flock (Figure S2) as well as for an unobserved flock (see Figure 1 in the main text), representing the overall prediction from the model for any healthy flock.

```
pred_df <-
  cbind(
    dat_healthy,
    100 * merTools::predictInterval(
      final_mod,
      newdata = dat_healthy, type = "probability", level = 0.95, n.sims = 10^5
    )
  )
```

```r
pred_df1 <-
  data.frame(
    id = "FRNI",
    age = seq_len(max(dat_healthy$age)),
    popsize = 1
  )
pred_df1 <-
  cbind(
    pred_df1,
    100 * merTools::predictInterval(
      final_mod,
      newdata = pred_df1, type = "probability", level = 0.95, n.sims = 10^5
    )
  )
```

The upper bound of the 95% prediction interval was considered as the maximum daily mortality expected in a non-HPAI-infected flock [7, 9, 10], and was used to define fixed reporting thresholds whose performances were evaluated and compared against the already existing thresholds. To define those thresholds, we used the median, the third quartile and the maximum value of the upper bound of the 95% prediction interval: 0.11%, 0.14%, 0.35%.

2.2 Mortality above a given threshold for a single day

We tested five mortality thresholds : the three defined above (see 2.1), as well as the 0.25% and 2% thresholds that were defined in the French legislation. An alarm was defined as an increase in mortality above the mortality threshold at any given day.

As for healthy flocks, we first calculated the percentage of death that occurred every day for infected flocks:

```r
dat_infected$popsize <- unlist(sapply(levels(dat_infected$id), function(j) {
  flocks$size[flocks$id == j] -
    c(0, cumsum(dat_infected$mortality[dat_infected$id == j])[
      -sum(dat_infected$id == j)
    ])
}), use.names = FALSE)

dat_infected$propmort <- dat_infected$mortality / dat_infected$popsize
```

With the data from infected flocks, we calculated the sensitivity (Se = number of infected flocks where an alarm was raised/total number of infected flocks) and the timeliness (T = time where an alarm was raised when using the proposed thresholds compared with the time where an alarm would have been raised using the 0.25% threshold).

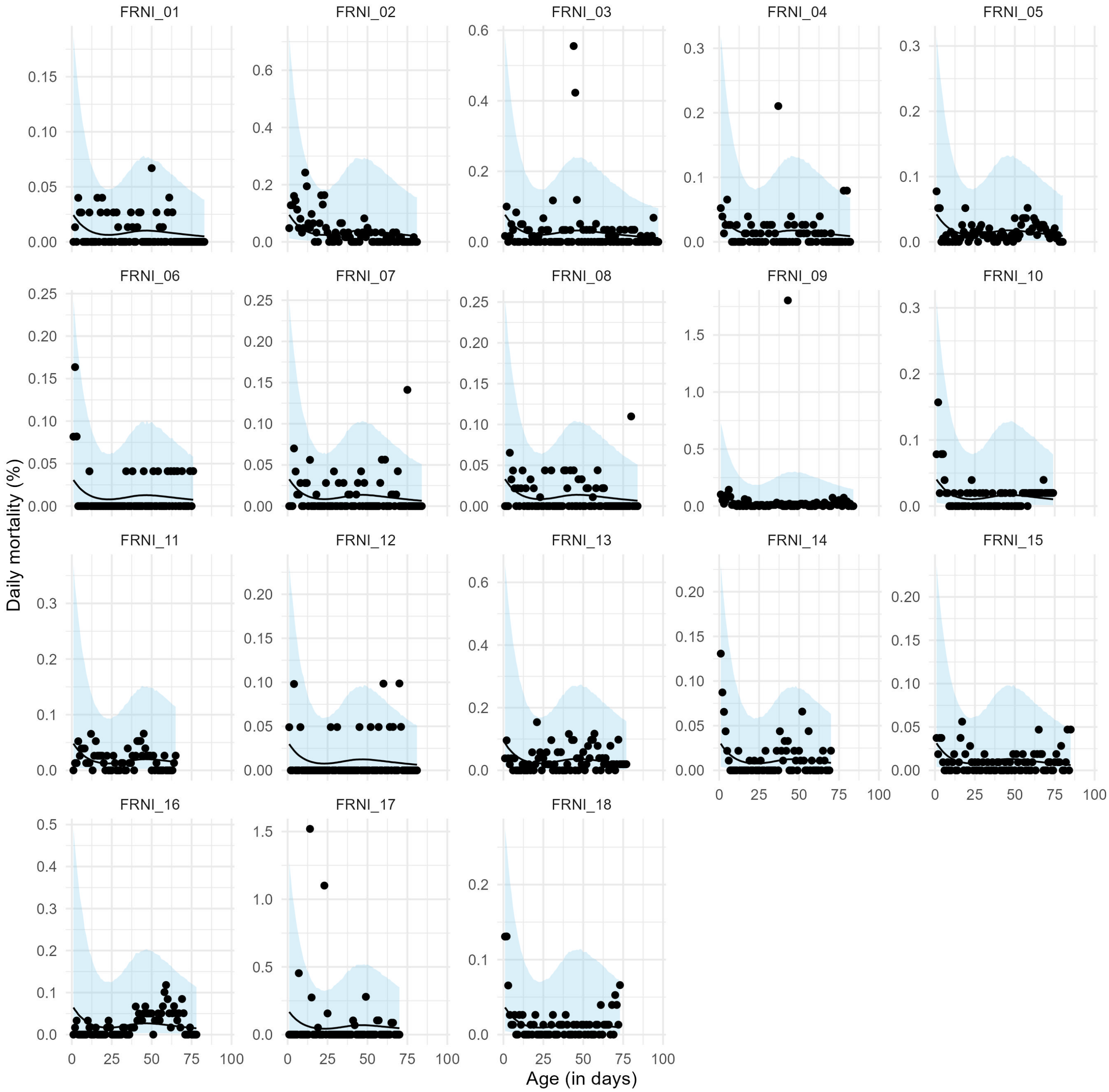

Figure S2: Predictions from the generalized linear mixed model showing the estimated expected daily mortality (%) and 95% prediction interval in 18 healthy mule duck flocks.

```r
thrs <-
  sort(c(round(quantile(pred_df1$upr, names = FALSE)[-c(1, 2)], 2), 0.25, 2))
names(thrs) <- paste0(thrs, "%")

se <-
  sapply(thrs, function(i) {
    sum(sapply(levels(dat_infected$id), function(j) {
      any(dat_infected$propmort[dat_infected$id == j] >= (i / 100))
    }))
  })

t <-
  sapply(thrs, function(i) {
    sapply(levels(dat_infected$id), function(j) {
      dat_infected$age[dat_infected$id == j][
        max(dat_infected$age[dat_infected$id == j]) - 10 +
          match(TRUE, dat_infected$propmort[dat_infected$id == j][
            dat_infected$age[dat_infected$id == j] >=
              max(dat_infected$age[dat_infected$id == j]) - 10
          ] >= (0.25 / 100)) - 1
      ] -
        dat_infected$age[dat_infected$id == j][
          max(dat_infected$age[dat_infected$id == j]) - 10 +
            match(TRUE, dat_infected$propmort[dat_infected$id == j][
              dat_infected$age[dat_infected$id == j] >=
                max(dat_infected$age[dat_infected$id == j]) - 10
            ] >= (i / 100)) - 1
        ]
    })
  })
```

Among the 12 HPAI-infected flocks, 12, 12, 12, 12, 8 were detected for the 0.11%, 0.14%, 0.25%, 0.35%, 2% thresholds, respectively. Timeliness was similar or better for thresholds lower than 0.25%, while thresholds above performed similar or worse:

```r
summary(t, digits = 2)
```

```
##      0.11%        0.14%          0.25%        0.35%             2%
##  Min.   :0   Min.   :0.00   Min.   :0   Min.   :-1.00   Min.   :-3.00
##  1st Qu.:0   1st Qu.:0.00   1st Qu.:0   1st Qu.:-1.00   1st Qu.:-1.25
##  Median :1   Median :0.00   Median :0   Median : 0.00   Median :-1.00
##  Mean   :1   Mean   :0.42   Mean   :0   Mean   :-0.33   Mean   :-1.12
##  3rd Qu.:2   3rd Qu.:1.00   3rd Qu.:0   3rd Qu.: 0.00   3rd Qu.:-0.75
```

```
##  Max.   :3   Max.   :1.00   Max.   :0   Max.    : 0.00   Max.   : 0.00
##                                                           NA's   :4
```

With the data from healthy flocks, we calculated the specificity (Sp = number of healthy flocks with no alarm/total number of healthy flocks) and the number of false alarms per 100 days of production (FA).

```
sp <-
  length(levels(dat_healthy$id)) -
  sapply(thrs, function(i) {
    sum(sapply(levels(dat_healthy$id), function(j) {
      any(dat_healthy$propmort[dat_healthy$id == j] >= (i / 100))
    }))
  })

fa <-
  sapply(thrs, function(i) {
    sapply(levels(dat_healthy$id), function(j) {
      100 * sum(dat_healthy$propmort[dat_healthy$id == j] >= (i / 100)) /
        length(na.omit(dat_healthy$mortality[dat_healthy$id == j]))
    })
  })
```

Among the 18 healthy flocks, 6, 9, 15, 15, 18 had no alarm for the 0.11%, 0.14%, 0.25%, 0.35%, 2% thresholds, respectively. The number of false alarms decreased with increasing thresholds:

```
summary(fa, digits = 2)
```

```
##      0.11%          0.14%          0.25%          0.35%            2%
##  Min.   : 0.0   Min.   :0.0   Min.   :0.00   Min.   :0.00   Min.   :0
##  1st Qu.: 0.0   1st Qu.:0.0   1st Qu.:0.00   1st Qu.:0.00   1st Qu.:0
##  Median : 1.3   Median :0.6   Median :0.00   Median :0.00   Median :0
##  Mean   : 2.3   Mean   :1.5   Mean   :0.58   Mean   :0.42   Mean   :0
##  3rd Qu.: 2.7   3rd Qu.:1.3   3rd Qu.:0.00   3rd Qu.:0.00   3rd Qu.:0
##  Max.   :12.3   Max.   :8.6   Max.   :7.14   Max.   :4.29   Max.   :0
```

Figures S3 and S4 illustrate the performance of the 0.25% threshold.

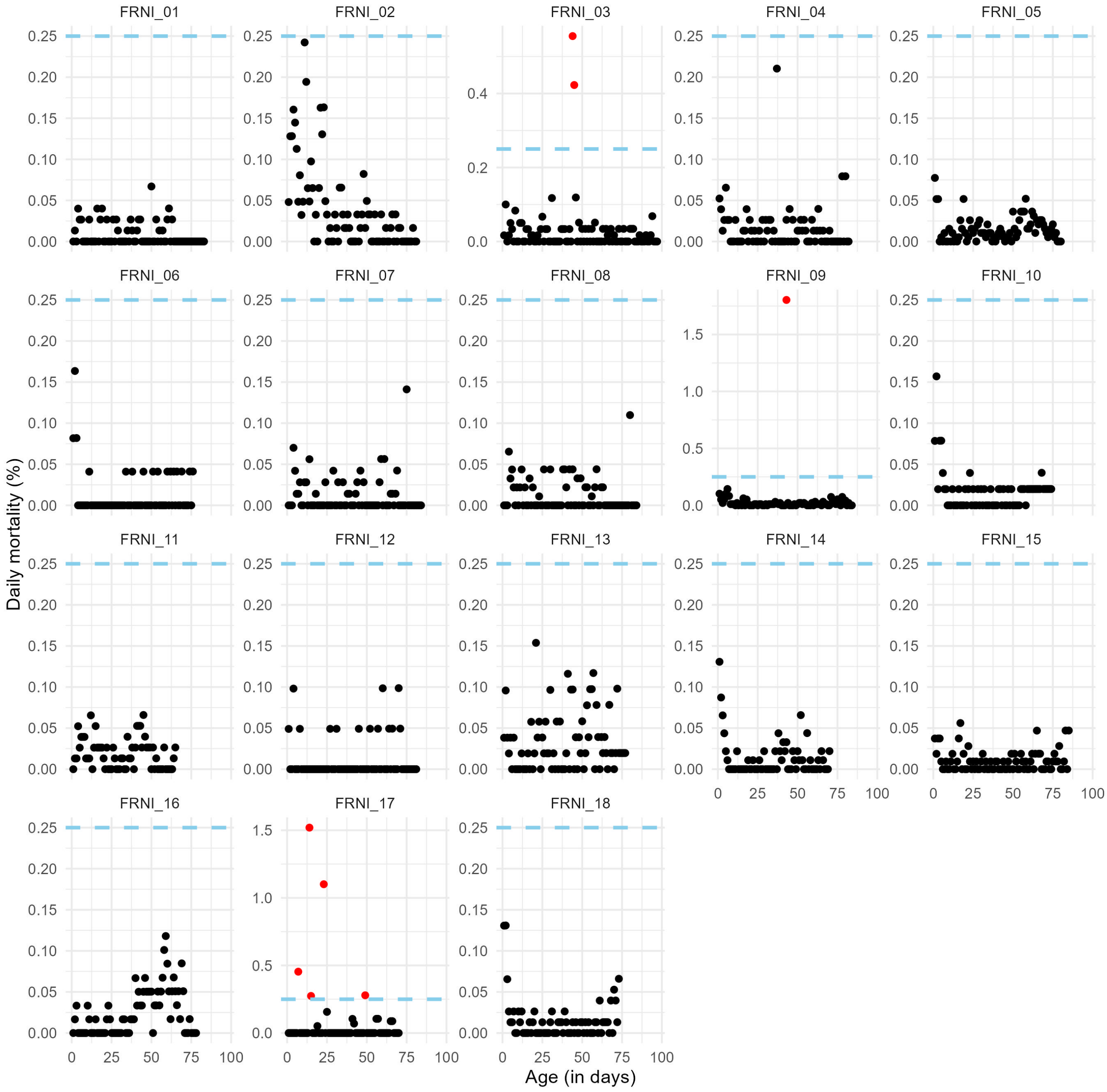

Figure S3: Illustration of the performance of the 0.25% fixed daily mortality threshold in healthy flocks. Red dots indicate when an alarm was raised (i.e. daily mortality above the threshold).

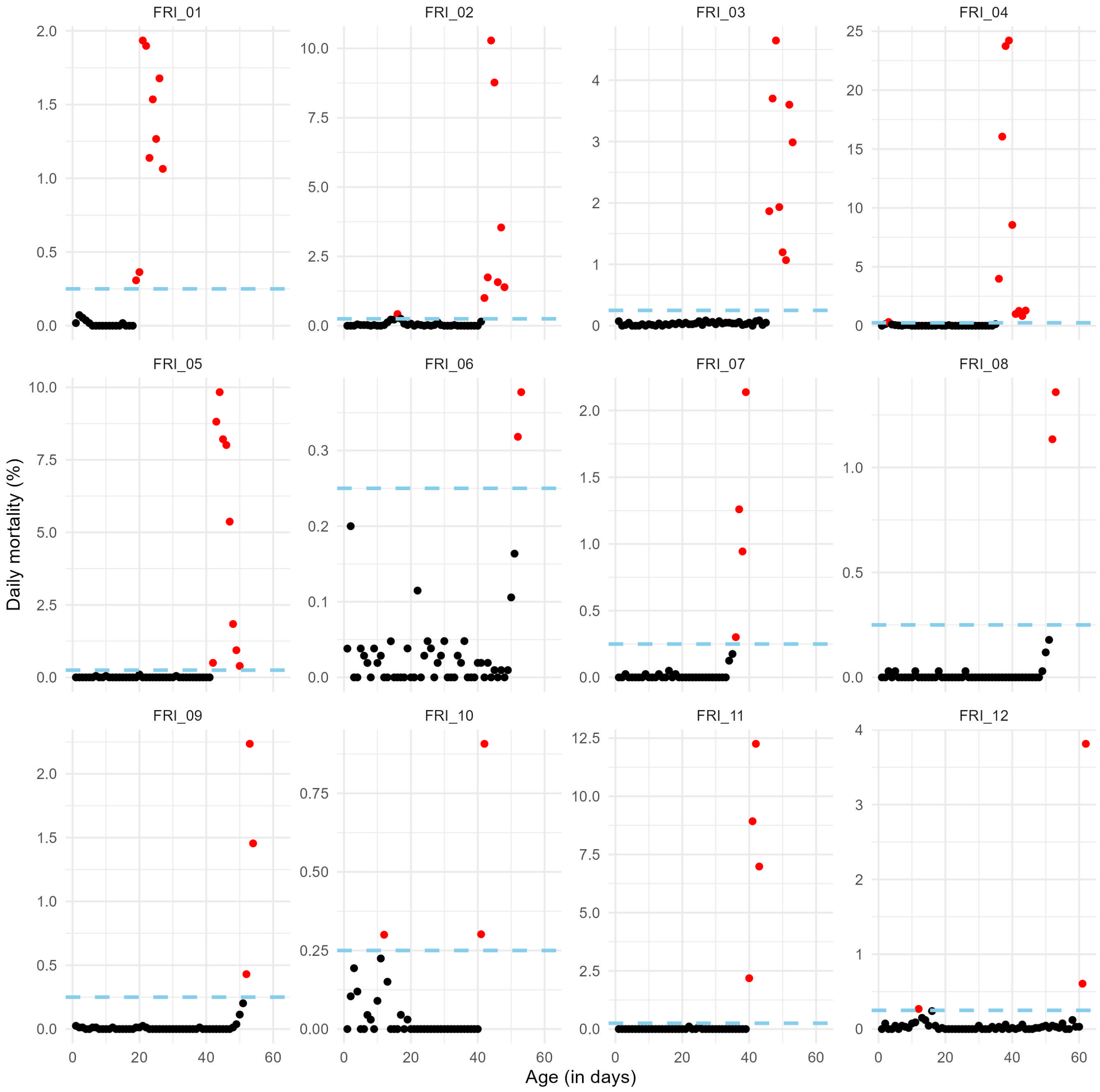

Figure S4: Illustration of the performance of the 0.25% fixed daily mortality threshold in HPAI-infected flocks. Red dots indicate when an alarm was raised (i.e. daily mortality above the threshold).

2.3 Mortality above a given threshold for two consecutive days

We tested the same thresholds as before, except for the highest (2%) as it was defined in the French legislation for a single day [13]. This time, an alarm was defined as an increase in mortality above the mortality threshold for two consecutive days.

Timeliness was defined as the time where an alarm was raised when using the proposed thresholds (over two consecutive days) compared with the time where an alarm would have been raised using the 0.25% threshold over a single day (for comparison purposes).

```
se2 <-
  sapply(thrs[-length(thrs)], function(i) {
    sum(sapply(levels(dat_infected$id), function(j) {
      any(
        (dat_infected$propmort[dat_infected$id == j] >= (i / 100)) &
          (c(FALSE, dat_infected$propmort[dat_infected$id == j][
            -sum(dat_infected$id == j)
          ] >= (i / 100)))
      )
    }))
  })

t2 <-
  sapply(thrs[-length(thrs)], function(i) {
    sapply(levels(dat_infected$id), function(j) {
      dat_infected$age[dat_infected$id == j][
        max(dat_infected$age[dat_infected$id == j]) - 10 +
          match(TRUE, dat_infected$propmort[dat_infected$id == j][
            dat_infected$age[dat_infected$id == j] >=
              max(dat_infected$age[dat_infected$id == j]) - 10
          ] >= (0.25 / 100)) - 1
      ] -
        dat_infected$age[dat_infected$id == j][
          max(dat_infected$age[dat_infected$id == j]) - 10 +
            match(
              TRUE,
              dat_infected$propmort[dat_infected$id == j][
                dat_infected$age[dat_infected$id == j] >=
                  max(dat_infected$age[dat_infected$id == j]) - 10
              ] >= (i / 100) &
                dat_infected$propmort[dat_infected$id == j][
                  dat_infected$age[dat_infected$id == j] >=
                    max(dat_infected$age[dat_infected$id == j]) - 11 &
                    dat_infected$age[dat_infected$id == j] <
                      max(dat_infected$age[dat_infected$id == j])
```

```
                ] >= (i / 100)
            ) - 1
        ]
    })
  })
```

Among the 12 HPAI-infected flocks, 12, 12, 12, 10 were detected for the 0.11%, 0.14%, 0.25%, 0.35% thresholds, respectively. Timeliness was usually worse when using thresholds for two consecutive days compared to the one-day 0.25% threshold:

```
summary(t2, digits = 2)
```

```
##      0.11%           0.14%           0.25%          0.35%
##  Min.   :-1.00   Min.   :-1.00   Min.   :-1   Min.   :-2.0
##  1st Qu.:-1.00   1st Qu.:-1.00   1st Qu.:-1   1st Qu.:-1.0
##  Median :-0.50   Median :-1.00   Median :-1   Median :-1.0
##  Mean   :-0.25   Mean   :-0.58   Mean   :-1   Mean   :-1.2
##  3rd Qu.: 0.25   3rd Qu.: 0.00   3rd Qu.:-1   3rd Qu.:-1.0
##  Max.   : 1.00   Max.   : 0.00   Max.   :-1   Max.   :-1.0
##                                               NA's   :2
```

```
sp2 <-
  length(levels(dat_healthy$id)) -
  sapply(thrs[-length(thrs)], function(i) {
    sum(sapply(levels(dat_healthy$id), function(j) {
      any(
        (dat_healthy$propmort[dat_healthy$id == j] >= (i / 100)) &
          (c(FALSE, dat_healthy$propmort[dat_healthy$id == j][
            -sum(dat_healthy$id == j)
          ] >= (i / 100)))
      )
    }))
  })

fa2 <-
  sapply(thrs[-length(thrs)], function(i) {
    sapply(levels(dat_healthy$id), function(j) {
      100 * sum(
        (dat_healthy$propmort[dat_healthy$id == j] >= (i / 100)) &
          (c(FALSE, dat_healthy$propmort[dat_healthy$id == j][
            -sum(dat_healthy$id == j)
          ] >= (i / 100)))
      ) /
```

```r
      length(na.omit(dat_healthy$mortality[dat_healthy$id == j]))
    })
  })
```

Among the 18 healthy flocks, 14, 15, 16, 17 had no alarm for the 0.11%, 0.14%, 0.25%, 0.35% thresholds, respectively. The number of false alarms per 100 days of production decreased with increasing thresholds:

```r
summary(fa2, digits = 2)
```

```
##      0.11%           0.14%           0.25%           0.35%
##  Min.   :0.00   Min.   :0.00   Min.   :0.00   Min.   :0.000
##  1st Qu.:0.00   1st Qu.:0.00   1st Qu.:0.00   1st Qu.:0.000
##  Median :0.00   Median :0.00   Median :0.00   Median :0.000
##  Mean   :0.75   Mean   :0.27   Mean   :0.14   Mean   :0.057
##  3rd Qu.:0.00   3rd Qu.:0.00   3rd Qu.:0.00   3rd Qu.:0.000
##  Max.   :8.64   Max.   :2.47   Max.   :1.43   Max.   :1.031
```

2.4 Mortality that doubles after being above a given threshold

We tested the same thresholds as before. This time, an alarm was defined as an increase in mortality above the mortality threshold at any given day, followed the next day by at least the doubling of the mortality.

Timeliness was defined as the time where an alarm was raised when using the proposed thresholds (over two consecutive days) compared with the time where an alarm would have been raised using the 0.25% threshold over a single day (for comparison purposes).

```r
se3 <-
  sapply(thrs[-length(thrs)], function(i) {
    sum(sapply(levels(dat_infected$id), function(j) {
      any(c(
        FALSE,
        dat_infected$propmort[dat_infected$id == j][-1] >
          (2 * dat_infected$propmort[dat_infected$id == j][
            -sum(dat_infected$id == j)
          ])
      ) &
        (dat_infected$propmort[dat_infected$id == j] >= (i / 100)) &
        (c(FALSE, dat_infected$propmort[dat_infected$id == j][
          -sum(dat_infected$id == j)
        ] >= (i / 100))))
```

```
    }))
  })

t3 <-
  sapply(thrs[-length(thrs)], function(i) {
    sapply(levels(dat_infected$id), function(j) {
      dat_infected$age[dat_infected$id == j][
        max(dat_infected$age[dat_infected$id == j]) - 10 +
          match(TRUE, dat_infected$propmort[dat_infected$id == j][
            dat_infected$age[dat_infected$id == j] >=
              max(dat_infected$age[dat_infected$id == j]) - 10
          ] >= (0.25 / 100)) - 1
      ] -
        dat_infected$age[dat_infected$id == j][
          max(dat_infected$age[dat_infected$id == j]) - 10 +
            match(
              TRUE,
              dat_infected$propmort[dat_infected$id == j][
                dat_infected$age[dat_infected$id == j] >=
                  max(dat_infected$age[dat_infected$id == j]) - 10
              ] >= (i / 100) &
                dat_infected$propmort[dat_infected$id == j][
                  dat_infected$age[dat_infected$id == j] >=
                    max(dat_infected$age[dat_infected$id == j]) - 11 &
                    dat_infected$age[dat_infected$id == j] <
                      max(dat_infected$age[dat_infected$id == j])
                ] >= (i / 100) &
                dat_infected$propmort[dat_infected$id == j][
                  dat_infected$age[dat_infected$id == j] >=
                    max(dat_infected$age[dat_infected$id == j]) - 10
                ] > 2 * dat_infected$propmort[dat_infected$id == j][
                  dat_infected$age[dat_infected$id == j] >=
                    max(dat_infected$age[dat_infected$id == j]) - 11 &
                    dat_infected$age[dat_infected$id == j] <
                      max(dat_infected$age[dat_infected$id == j])
                ]
            ) - 1
        ]
    })
  })
```

Among the 12 HPAI-infected flocks, 11, 11, 10, 9 were detected for the 0.11%, 0.14%, 0.25%, 0.35% thresholds, respectively. Timeliness was usually worse when using thresholds for two consecutive days compared to the one-day 0.25% threshold:

```r
summary(t3, digits = 2)
```

```
##      0.11%            0.14%            0.25%            0.35%     
##  Min.   :-6.0   Min.   :-6.0   Min.   :-6.0   Min.   :-6  
##  1st Qu.:-1.0   1st Qu.:-1.0   1st Qu.:-1.8   1st Qu.:-2  
##  Median :-1.0   Median :-1.0   Median :-1.0   Median :-1  
##  Mean   :-1.2   Mean   :-1.3   Mean   :-1.7   Mean   :-2  
##  3rd Qu.: 0.0   3rd Qu.:-0.5   3rd Qu.:-1.0   3rd Qu.:-1  
##  Max.   : 0.0   Max.   : 0.0   Max.   :-1.0   Max.   :-1  
##  NA's   :1      NA's   :1      NA's   :2      NA's   :3
```

```r
sp3 <-
  length(levels(dat_healthy$id)) -
  sapply(thrs[-length(thrs)], function(i) {
    sum(sapply(levels(dat_healthy$id), function(j) {
      any(c(
        FALSE,
        dat_healthy$propmort[dat_healthy$id == j][-1] >
          (2 * dat_healthy$propmort[dat_healthy$id == j][
            -sum(dat_healthy$id == j)
          ])
      ) &
        (dat_healthy$propmort[dat_healthy$id == j] >= (i / 100)) &
        (c(FALSE, dat_healthy$propmort[dat_healthy$id == j][
          -sum(dat_healthy$id == j)
        ] >= (i / 100))))
    }))
  })

fa3 <-
  sapply(thrs[-length(thrs)], function(i) {
    sapply(levels(dat_healthy$id), function(j) {
      100 * sum(c(
        FALSE,
        dat_healthy$propmort[dat_healthy$id == j][-1] >
          (2 * dat_healthy$propmort[dat_healthy$id == j][
            -sum(dat_healthy$id == j)
          ])
      ) &
        (dat_healthy$propmort[dat_healthy$id == j] >= (i / 100)) &
        (c(FALSE, dat_healthy$propmort[dat_healthy$id == j][
          -sum(dat_healthy$id == j)
        ] >= (i / 100)))) /
```

```
      length(na.omit(dat_healthy$mortality[dat_healthy$id == j]))
    })
  })
```

Among the 18 healthy flocks, 18, 18, 18, 18 had no alarm for the 0.11%, 0.14%, 0.25%, 0.35% thresholds, respectively.

3 Mortality ratio

3.1 Expected baseline mortality mortality ratio

In addition to fixed mortality thresholds, we also evaluated thresholds for the mortality ratio developed by Gonzales et al. [7]. This ratio has the benefit of adjusting to a flock specific mortality, as it uses mortality data from the previous week from the same flock. It is also simple and easy to apply for farmers, as it can be manually written and calculated on production calendars in paper form [7]. This ratio is calculated daily by [7]:

$$R_{ij} = \frac{d_{ij}}{M_{j-1}} \tag{1}$$

where R_{ij} is the mortality ratio of day i (1, ..., 7) of week j, d_{ij} is the number of ducks that died at day i in week j and M_{j-1} is the average mortality the previous week (sum of daily mortality/7).

When M_{j-1} was null, its value was replaced by the average mortality of the previous week with non-zero mortality value.

To define reference thresholds, a similar approach to that taken for the fixed daily mortality threshold was taken. We fitted a GLMM where the daily number of dead ducks was the response variable, the age of ducks (in days) was the explanatory variable, the natural logarithm of M_{j-1} the offset, and the flock identifier was the random effect [7]. To define the ratio thresholds, we used the median, the third quartile and the maximum value of the upper bound of the 95% prediction interval.

```
dat_healthy$avg7 <-
  unlist(sapply(levels(dat_healthy$id), function(j) {
    c(
      rep(NA, 7),
      rep(zoo::rollmean(dat_healthy$mortality[dat_healthy$id ==
        j], 7, fill = NA, align = "right")[seq(
        7,
        sum(dat_healthy$id == j), 7
      )], each = 7)
    )[seq_len(sum(dat_healthy$id == j))]
```

```r
  }), use.names = FALSE)

# Replace null values of avg7
for (i in seq_len(nrow(dat_healthy))) {
  x <- dat_healthy$avg7[dat_healthy$age == i]
  dat_healthy$avg7[dat_healthy$age == i] <-
    ifelse(x == 0,
      dat_healthy$avg7[dat_healthy$age == i - 1],
      x
    )
}

dat_healthy$ratio <- dat_healthy$mortality / dat_healthy$avg7

mod_pois2 <-
  lme4::glmer(mortality ~ age + (1 | id) + offset(log(avg7)),
    data = dat_healthy, family = poisson
  )

mod_nb2 <-
  lme4::glmer.nb(mortality ~ age + (1 | id) + offset(log(avg7)),
    data = dat_healthy
  )

aic2 <- rep(0, 6)
for (df in seq_len(length(aic2))) {
  aic2[df] <- AIC(
    lme4::glmer.nb(
      mortality ~ splines::ns(age, df) + (1 | id) + offset(log(avg7)),
      data = dat_healthy
    )
  )
}

final_mod2 <-
  lme4::glmer.nb(
    mortality ~
      splines::ns(age, which.min(aic2)) + (1 | id) + offset(log(avg7)),
    data = dat_healthy
  )

pred_df2 <-
  data.frame(
    id = "FRNI",
```

```r
    age = seq(8, max(dat_healthy$age), 1),
    avg7 = 1
  )
pred_df2 <-
  cbind(
    pred_df2,
    merTools::predictInterval(
      final_mod2,
      newdata = pred_df2, type = "probability", level = 0.95, n.sims = 10^5
    )
  )

thrs2 <- sort(c(round(quantile(pred_df2$upr, names = FALSE)[-c(1, 2)], 1)))
thrs2 <- c(thrs2, 2 * max(thrs2))
names(thrs2) <- paste0(thrs2)

dat_infected$avg7 <-
  unlist(sapply(levels(dat_infected$id), function(j) {
    c(
      rep(NA, 7),
      rep(zoo::rollmean(dat_infected$mortality[dat_infected$id ==
        j], 7, fill = NA, align = "right")[seq(
        7,
        sum(dat_infected$id == j), 7
      )], each = 7)
    )[seq_len(sum(dat_infected$id == j))]
  }), use.names = FALSE)

# Replace null values of avg7
for (i in seq_len(nrow(dat_infected))) {
  x <- dat_infected$avg7[dat_infected$age == i]
  dat_infected$avg7[dat_infected$age == i] <-
    ifelse(x == 0,
      dat_infected$avg7[dat_infected$age == i - 1],
      x
    )
}

dat_infected$ratio <- dat_infected$mortality / dat_infected$avg7
```

3.2 Mortality ratio above a given threshold for a single day

We tested four ratio thresholds : the three defined above (see 3.1), as well as twice the maximum value of the upper bound of the 95% prediction interval. An alarm was defined as an increase in the mortality ratio above the ratio threshold at any given day [8].

```
se4 <-
  sapply(thrs2, function(i) {
    sum(sapply(levels(dat_infected$id), function(j) {
      any(dat_infected$ratio[dat_infected$id == j] >= i, na.rm = TRUE)
    }))
  })

t4 <-
  sapply(thrs2, function(i) {
    sapply(levels(dat_infected$id), function(j) {
      dat_infected$age[dat_infected$id == j][
        max(dat_infected$age[dat_infected$id == j]) - 10 +
          match(TRUE, dat_infected$propmort[dat_infected$id == j][
            dat_infected$age[dat_infected$id == j] >=
              max(dat_infected$age[dat_infected$id == j]) - 10
          ] >= (0.25 / 100)) - 1
      ] -
        dat_infected$age[dat_infected$id == j][
          max(dat_infected$age[dat_infected$id == j]) - 10 +
            match(TRUE, dat_infected$ratio[dat_infected$id == j][
              dat_infected$age[dat_infected$id == j] >=
                max(dat_infected$age[dat_infected$id == j]) - 10
            ] >= i) - 1
        ]
    })
  })
```

Among the 12 HPAI-infected flocks, 12, 12, 12, 12 were detected for ratio thresholds of 8, 10.7, 17.2, 34.4, respectively. Timeliness was usually better when using the mortality ratio compared to the one-day 0.25% threshold, except for the highest ratio threshold:

```
summary(t4, digits = 2)
```

```
##        8              10.7            17.2              34.4
##  Min.   :0.00   Min.   :0.00   Min.   :-2.00   Min.   :-3.0
##  1st Qu.:0.00   1st Qu.:0.00   1st Qu.: 0.00   1st Qu.:-1.0
##  Median :0.50   Median :0.00   Median : 0.00   Median : 0.0
##  Mean   :0.92   Mean   :0.83   Mean   : 0.33   Mean   :-0.5
```

```
##   3rd Qu.:2.00   3rd Qu.:2.00   3rd Qu.: 1.00   3rd Qu.: 0.0
##   Max.   :3.00   Max.   :3.00   Max.   : 3.00   Max.   : 1.0
```

```
sp4 <-
  length(levels(dat_healthy$id)) -
  sapply(thrs2, function(i) {
    sum(sapply(levels(dat_healthy$id), function(j) {
      any(dat_healthy$ratio[dat_healthy$id == j] >= i, na.rm = TRUE)
    }))
  })

fa4 <-
  sapply(thrs2, function(i) {
    sapply(levels(dat_healthy$id), function(j) {
      100 * sum(dat_healthy$ratio[dat_healthy$id == j] >= i, na.rm = TRUE) /
        length(na.omit(dat_healthy$ratio[dat_healthy$id == j]))
    })
  })
```

Among the 18 healthy flocks, 4, 5, 13, 16 had no alarm for ratio thresholds of 8, 10.7, 17.2, 34.4, respectively. The number of false alarms per 100 days of production decreased with increasing thresholds:

```
summary(fa4, digits = 2)
```

```
##         8             10.7            17.2            34.4
##   Min.   :0.0   Min.   :0.00   Min.   :0.00   Min.   :0.00
##   1st Qu.:1.3   1st Qu.:0.32   1st Qu.:0.00   1st Qu.:0.00
##   Median :2.6   Median :1.36   Median :0.00   Median :0.00
##   Mean   :2.4   Mean   :1.67   Mean   :0.60   Mean   :0.15
##   3rd Qu.:3.8   3rd Qu.:2.76   3rd Qu.:0.97   3rd Qu.:0.00
##   Max.   :5.3   Max.   :4.76   Max.   :3.17   Max.   :1.43
```

Figures S5 and S6 illustrate the performance of the 34.4 ratio threshold.

3.3 Mortality ratio above a given threshold for two consecutive days

Originally, the performance of the mortality ratio was evaluated when the threshold was exceeded for two consecutive days [7]. Therefore, this time an alarm was defined as an increase in the mortality ratio above the ratio threshold for two consecutive days.

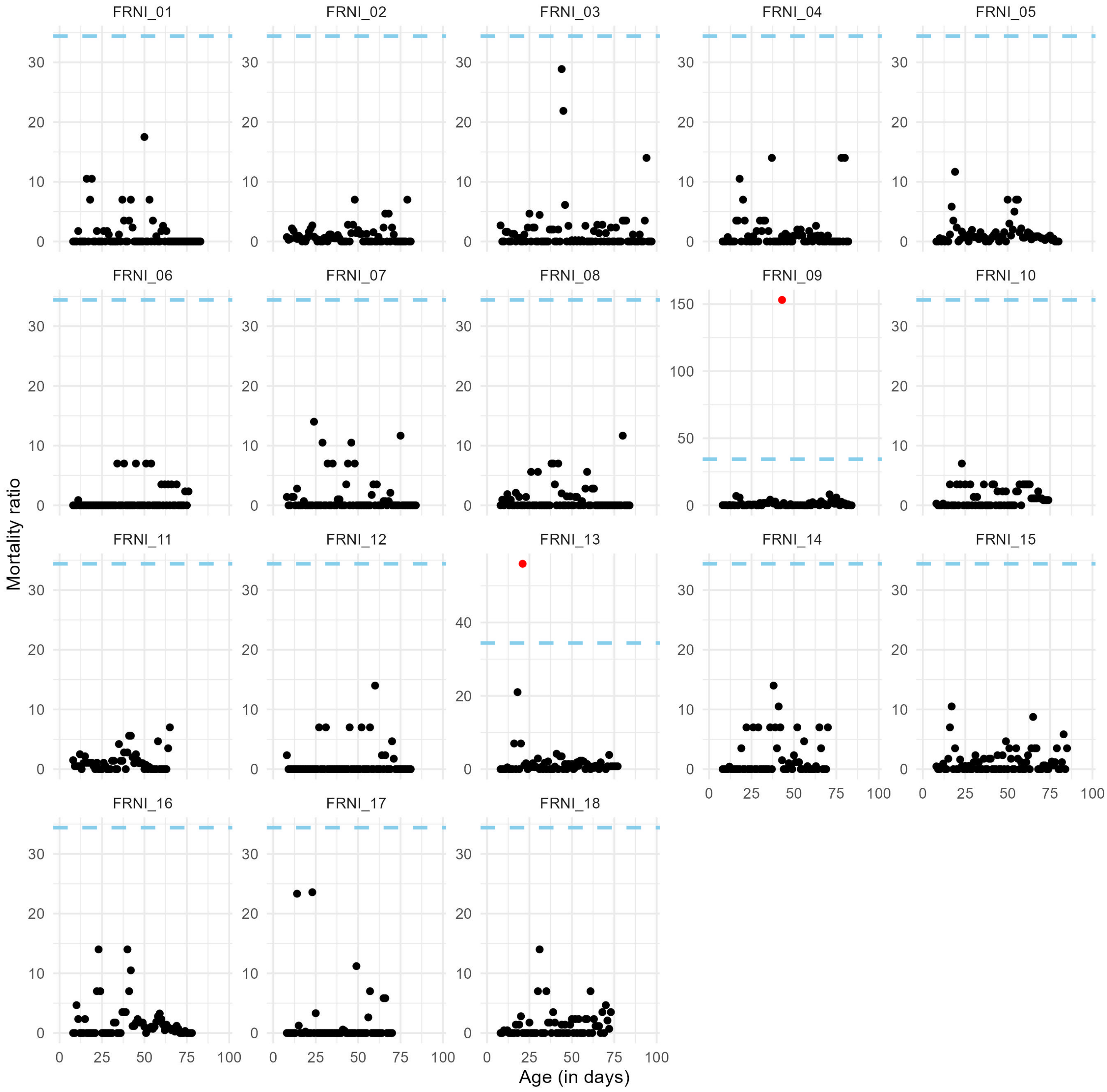

Figure S5: Illustration of the performance of the mortality ratio in healthy flocks. Red dots indicate when an alarm was raised (i.e. daily mortality above the threshold).

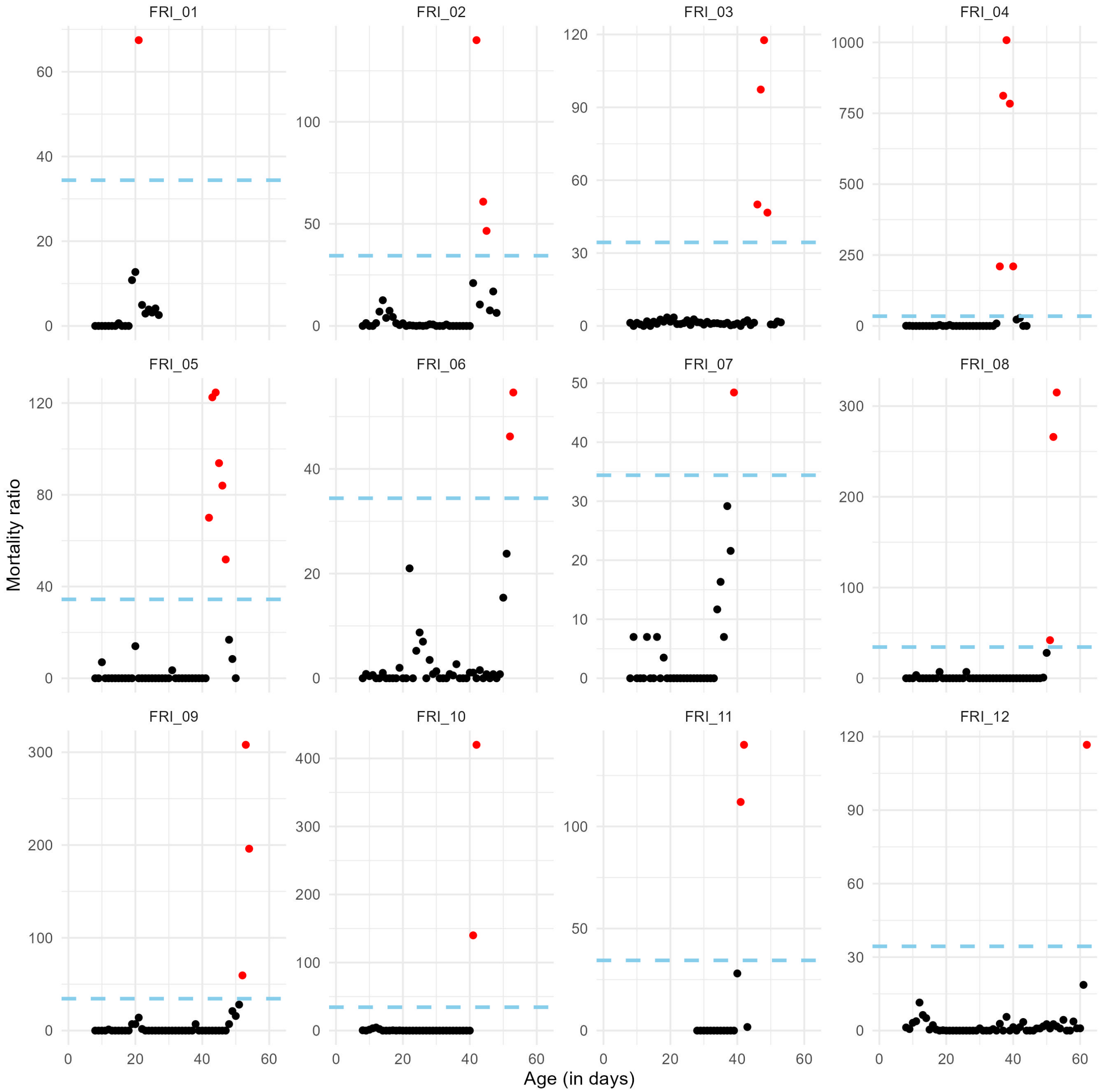

Figure S6: Illustration of the performance of the mortality ratio in HPAI-infected flocks. Red dots indicate when an alarm was raised (i.e. daily mortality above the threshold).

```r
se5 <-
  sapply(thrs2, function(i) {
    sum(sapply(levels(dat_infected$id), function(j) {
      any(
        dat_infected$ratio[dat_infected$id == j] >= i &
          c(FALSE, dat_infected$ratio[dat_infected$id == j][
            -sum(dat_infected$id == j)
          ] >= i),
        na.rm = TRUE
      )
    }))
  })

t5 <-
  sapply(thrs2, function(i) {
    sapply(levels(dat_infected$id), function(j) {
      dat_infected$age[dat_infected$id == j][
        max(dat_infected$age[dat_infected$id == j]) - 10 +
          match(TRUE, dat_infected$propmort[dat_infected$id == j][
            dat_infected$age[dat_infected$id == j] >=
              max(dat_infected$age[dat_infected$id == j]) - 10
          ] >= (0.25 / 100)) - 1
      ] -
        dat_infected$age[dat_infected$id == j][
          max(dat_infected$age[dat_infected$id == j]) - 10 +
            match(
              TRUE,
              dat_infected$ratio[dat_infected$id == j][
                dat_infected$age[dat_infected$id == j] >=
                  max(dat_infected$age[dat_infected$id == j]) - 10
              ] >= i &
                dat_infected$ratio[dat_infected$id == j][
                  dat_infected$age[dat_infected$id == j] >=
                    max(dat_infected$age[dat_infected$id == j]) - 11 &
                    dat_infected$age[dat_infected$id == j] <
                      max(dat_infected$age[dat_infected$id == j])
                ] >= i
            ) - 1
        ]
    })
  })
```

Among the 12 HPAI-infected flocks, 12, 12, 11, 9 were detected for ratio thresholds of 8, 10.7, 17.2, 34.4, respectively. Timeliness was usually worse when using the mortality ratio compared to the

one-day 0.25% threshold:

```
summary(t5, digits = 2)
```

```
##        8               10.7            17.2            34.4
##  Min.   :-1.000   Min.   :-1.00   Min.   :-2.00   Min.   :-3.0
##  1st Qu.:-1.000   1st Qu.:-1.00   1st Qu.:-1.00   1st Qu.:-1.0
##  Median :-0.500   Median :-1.00   Median :-1.00   Median :-1.0
##  Mean   :-0.083   Mean   :-0.17   Mean   :-0.64   Mean   :-1.2
##  3rd Qu.: 1.000   3rd Qu.: 1.00   3rd Qu.: 0.00   3rd Qu.:-1.0
##  Max.   : 2.000   Max.   : 2.00   Max.   : 1.00   Max.   : 0.0
##                                   NA's   :1       NA's   :3
```

```
sp5 <-
  length(levels(dat_healthy$id)) -
  sapply(thrs2, function(i) {
    sum(sapply(levels(dat_healthy$id), function(j) {
      any(
        dat_healthy$ratio[dat_healthy$id == j] >= i &
          c(FALSE, dat_healthy$ratio[dat_healthy$id == j][
            -sum(dat_healthy$id == j)
          ] >= i),
        na.rm = TRUE
      )
    }))
  })

fa5 <-
  sapply(thrs2, function(i) {
    sapply(levels(dat_healthy$id), function(j) {
      100 * sum(
        dat_healthy$ratio[dat_healthy$id == j] >= i &
          c(FALSE, dat_healthy$ratio[dat_healthy$id == j][
            -sum(dat_healthy$id == j)
          ] >= i),
        na.rm = TRUE
      ) /
        length(na.omit(dat_healthy$ratio[dat_healthy$id == j]))
    })
  })
```

Among the 18 healthy flocks, 17, 17, 17, 18 had no alarm for ratio thresholds of 8, 10.7, 17.2, 34.4, respectively.

4 Seven-day moving-average

4.1 Thresholds for the seven-day moving-average trigger

In addition to fixed mortality thresholds and the mortality ratio, we also evaluated a seven-day moving-average [6]. As for the mortality ratio, the moving-average has the benefit of adjusting to a flock specific mortality, as it uses mortality data from the previous seven days from the same flock. An alarm is raised when the daily mortality on a given day i is greater than x times the average mortality of the past seven days (from day $i-7$ to day $i-1$). When the average mortality was null, its value was replaced by the previous non-zero average mortality.

To define reference thresholds, a similar approach to that taken before was taken. We fitted a GLMM where the daily number of dead ducks was the response variable, the age of ducks (in days) was the explanatory variable, the natural logarithm of the seven-day moving-average was the offset, and the flock identifier was the random effect. To define the thresholds x, we used the first quartile, the median, the third quartile and the maximum value of the upper bound of the 95% prediction interval.

```
dat_healthy$rollmean <-
  unlist(sapply(levels(dat_healthy$id), function(j) {
    c(
      NA,
      zoo::rollmean(dat_healthy$mortality[dat_healthy$id ==
        j], 7, fill = NA, align = "right")[-sum(dat_healthy$id == j)]
    )
  }), use.names = FALSE)

# Replace null values of rollmean
for (i in seq_len(nrow(dat_healthy))) {
  x <- dat_healthy$rollmean[dat_healthy$age == i]
  dat_healthy$rollmean[dat_healthy$age == i] <-
    ifelse(x == 0,
      dat_healthy$rollmean[dat_healthy$age == i - 1],
      x
    )
}

dat_healthy$rollmean_trigger <- dat_healthy$mortality / dat_healthy$rollmean

mod_pois3 <-
  lme4::glmer(mortality ~ age + (1 | id) + offset(log(rollmean)),
    data = dat_healthy, family = poisson
  )

mod_nb3 <-
```

```r
  lme4::glmer.nb(mortality ~ age + (1 | id) + offset(log(rollmean)),
    data = dat_healthy
  )

aic3 <- rep(0, 6)
for (df in seq_len(length(aic3))) {
  aic3[df] <- AIC(
    lme4::glmer.nb(
      mortality ~ splines::ns(age, df) + (1 | id) + offset(log(rollmean)),
      data = dat_healthy
    )
  )
}

final_mod3 <-
  lme4::glmer.nb(
    mortality ~
      splines::ns(age, which.min(aic3)) + (1 | id) + offset(log(rollmean)),
    data = dat_healthy
  )

pred_df3 <-
  data.frame(
    id = "FRNI",
    age = seq(8, max(dat_healthy$age), 1),
    rollmean = 1
  )
pred_df3 <-
  cbind(
    pred_df3,
    merTools::predictInterval(
      final_mod3,
      newdata = pred_df3, type = "probability", level = 0.95, n.sims = 10^5
    )
  )

thrs3 <- sort(c(round(quantile(pred_df3$upr, names = FALSE)[-1], 1)))
names(thrs3) <- paste0(thrs3)

dat_infected$rollmean <-
  unlist(sapply(levels(dat_infected$id), function(j) {
    c(
      NA,
      zoo::rollmean(dat_infected$mortality[dat_infected$id ==
```

```
        j], 7, fill = NA, align = "right")[-sum(dat_infected$id == j)]
    )
  }), use.names = FALSE)

# Replace null values of rollmean
for (i in seq_len(nrow(dat_infected))) {
  x <- dat_infected$rollmean[dat_infected$age == i]
  dat_infected$rollmean[dat_infected$age == i] <-
    ifelse(x == 0,
      dat_infected$rollmean[dat_infected$age == i - 1],
      x
    )
}

dat_infected$rollmean_trigger <- dat_infected$mortality / dat_infected$rollmean
```

4.2 Daily mortality above the moving-average for a single day

An alarm was triggered when the daily mortality at any given day was greater than 7.4, 8.6, 10.8, 14.1 times the average mortality of the past seven days.

```
se6 <-
  sapply(thrs3, function(i) {
    sum(sapply(levels(dat_infected$id), function(j) {
      any(dat_infected$rollmean_trigger[dat_infected$id == j] >= i,
        na.rm = TRUE
      )
    }))
  })

t6 <-
  sapply(thrs3, function(i) {
    sapply(levels(dat_infected$id), function(j) {
      dat_infected$age[dat_infected$id == j][
        max(dat_infected$age[dat_infected$id == j]) - 10 +
          match(TRUE, dat_infected$propmort[dat_infected$id == j][
            dat_infected$age[dat_infected$id == j] >=
              max(dat_infected$age[dat_infected$id == j]) - 10
          ] >= (0.25 / 100)) - 1
      ] -
        dat_infected$age[dat_infected$id == j][
          max(dat_infected$age[dat_infected$id == j]) - 10 +
            match(TRUE, dat_infected$rollmean_trigger[dat_infected$id == j][
```

```
            dat_infected$age[dat_infected$id == j] >=
              max(dat_infected$age[dat_infected$id == j]) - 10
          ] >= i) - 1
      ]
    })
  })
```

Among the 12 HPAI-infected flocks, 12, 12, 12, 12 were detected for thresholds of 7.4, 8.6, 10.8, 14.1 times the moving-average, respectively. Timeliness was usually better when using the seven-day moving-average compared to the one-day 0.25% threshold:

```
summary(t6, digits = 2)
```

```
##       7.4            8.6            10.8            14.1
##  Min.   :0.00   Min.   :0.00   Min.   :-1.00   Min.   :-1.00
##  1st Qu.:0.00   1st Qu.:0.00   1st Qu.: 0.00   1st Qu.: 0.00
##  Median :0.50   Median :0.50   Median : 0.00   Median : 0.00
##  Mean   :0.83   Mean   :0.83   Mean   : 0.67   Mean   : 0.67
##  3rd Qu.:1.25   3rd Qu.:1.25   3rd Qu.: 1.25   3rd Qu.: 1.25
##  Max.   :3.00   Max.   :3.00   Max.   : 3.00   Max.   : 3.00
```

```
sp6 <-
  length(levels(dat_healthy$id)) -
  sapply(thrs3, function(i) {
    sum(sapply(levels(dat_healthy$id), function(j) {
      any(dat_healthy$rollmean_trigger[dat_healthy$id == j] >= i, na.rm = TRUE)
    }))
  })

fa6 <-
  sapply(thrs3, function(i) {
    sapply(levels(dat_healthy$id), function(j) {
      100 * sum(dat_healthy$rollmean_trigger[dat_healthy$id == j] >= i,
        na.rm = TRUE
      ) /
        length(na.omit(dat_healthy$ratio[dat_healthy$id == j]))
    })
  })
```

Among the 18 healthy flocks, 6, 6, 8, 12 had no alarm for thresholds of 7.4, 8.6, 10.8, 14.1 times the moving-average, respectively. The number of false alarms per 100 days decreased with increasing thresholds:

```r
summary(fa6, digits = 2)
```

```
##       7.4            8.6            10.8           14.1      
##  Min.   :0.0   Min.   :0.0   Min.   :0.0   Min.   :0.00  
##  1st Qu.:0.0   1st Qu.:0.0   1st Qu.:0.0   1st Qu.:0.00  
##  Median :1.5   Median :1.4   Median :1.3   Median :0.00  
##  Mean   :1.9   Mean   :1.8   Mean   :1.3   Mean   :0.62  
##  3rd Qu.:3.6   3rd Qu.:2.7   3rd Qu.:2.1   3rd Qu.:1.25  
##  Max.   :5.2   Max.   :5.2   Max.   :4.8   Max.   :4.76
```

Figures S7 and S8 illustrate the performance of the 14.1 ratio threshold.

4.3 Daily mortality above the moving-average for two days

As before, we evaluated when the threshold was exceeded for two consecutive days. Therefore, this time an alarm was triggered when the daily mortality was greater than 7.4, 8.6, 10.8, 14.1 times the average mortality of the past seven days for two consecutive days.

```r
se7 <-
  sapply(thrs3, function(i) {
    sum(sapply(levels(dat_infected$id), function(j) {
      any(
        dat_infected$rollmean_trigger[dat_infected$id == j] >= i &
          c(FALSE, dat_infected$rollmean_trigger[dat_infected$id == j][
            -sum(dat_infected$id == j)
          ] >= i),
        na.rm = TRUE
      )
    }))
  })

t7 <-
  sapply(thrs3, function(i) {
    sapply(levels(dat_infected$id), function(j) {
      dat_infected$age[dat_infected$id == j][
        max(dat_infected$age[dat_infected$id == j]) - 10 +
          match(TRUE, dat_infected$propmort[dat_infected$id == j][
            dat_infected$age[dat_infected$id == j] >=
              max(dat_infected$age[dat_infected$id == j]) - 10
          ] >= (0.25 / 100)) - 1
      ] -
        dat_infected$age[dat_infected$id == j][
```

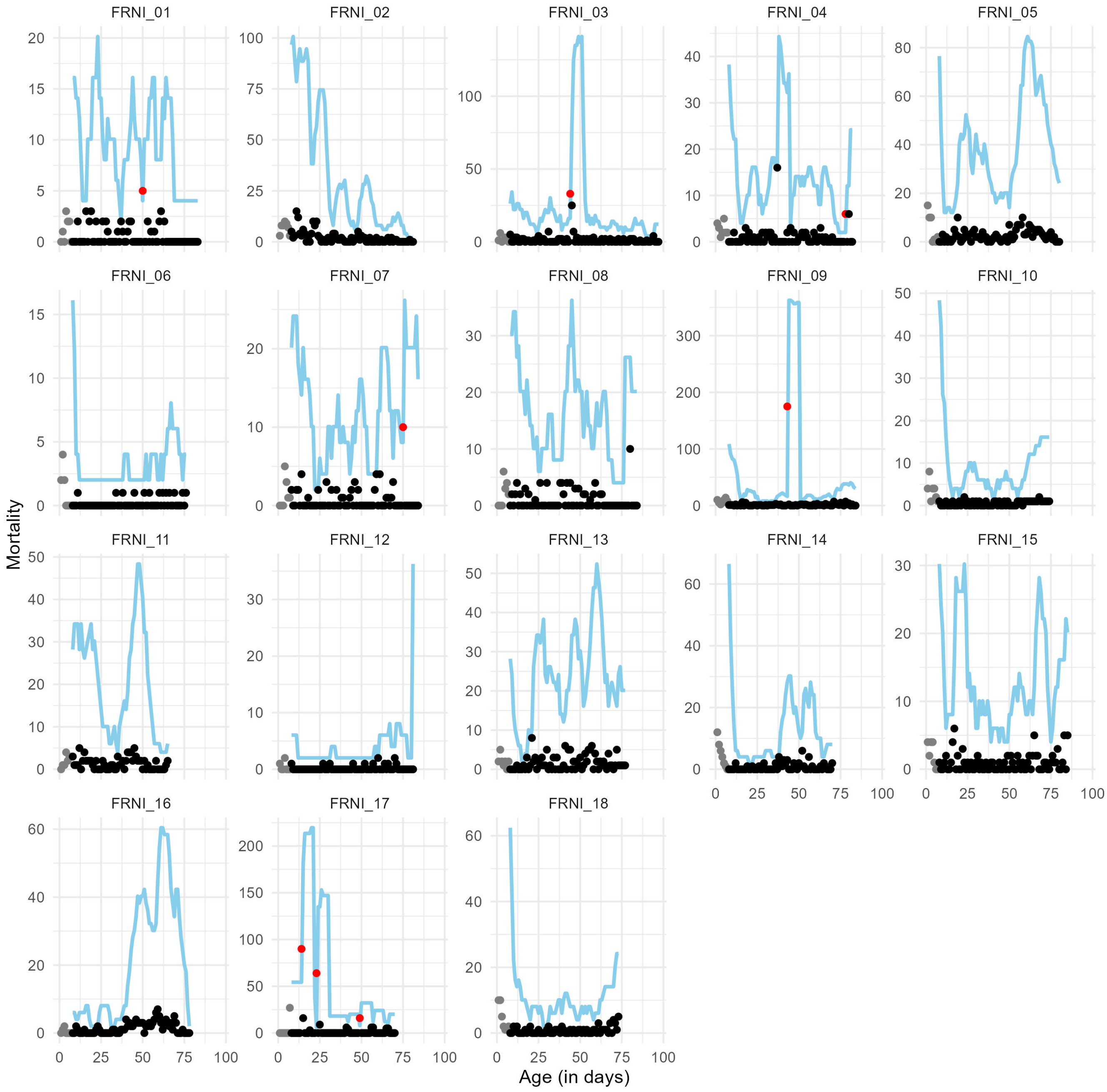

Figure S7: Illustration of the performance of the seven-day moving-average in healthy flocks. The blue line indicate the threshold of 14.1 times the moving-average. Red dots indicate when an alarm was raised (i.e. daily mortality above the threshold).

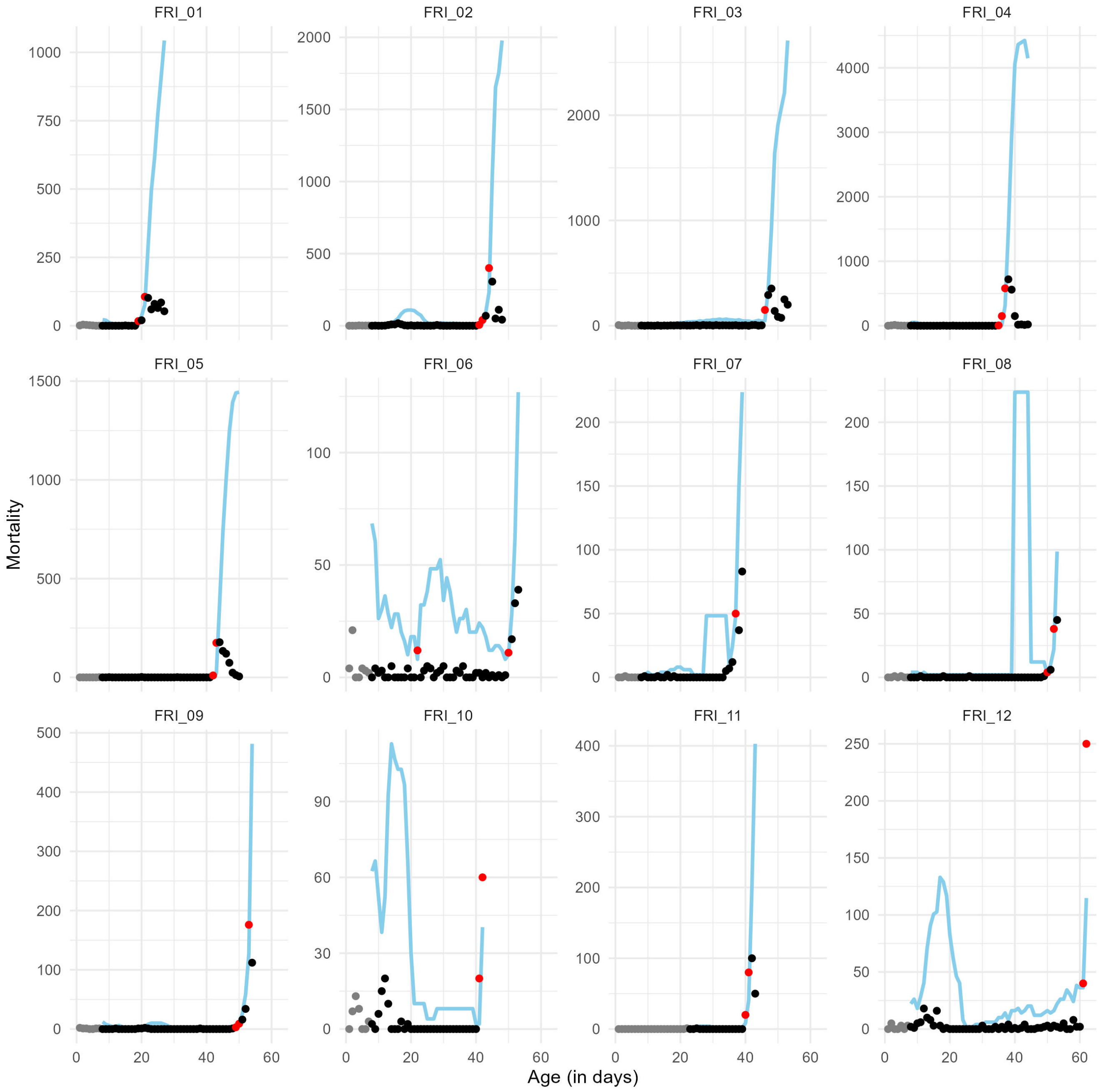

Figure S8: Illustration of the performance of the seven-day moving-average in HPAI-infected flocks. The blue line indicate the threshold of 14.1 times the moving-average. Red dots indicate when an alarm was raised (i.e. daily mortality above the threshold).

```r
          max(dat_infected$age[dat_infected$id == j]) - 10 +
            match(
              TRUE,
              dat_infected$rollmean_trigger[dat_infected$id == j][
                dat_infected$age[dat_infected$id == j] >=
                  max(dat_infected$age[dat_infected$id == j]) - 10
              ] >= i &
                dat_infected$rollmean_trigger[dat_infected$id == j][
                  dat_infected$age[dat_infected$id == j] >=
                    max(dat_infected$age[dat_infected$id == j]) - 11 &
                    dat_infected$age[dat_infected$id == j] <
                      max(dat_infected$age[dat_infected$id == j])
                ] >= i
            ) - 1
        ]
    })
  })
```

Among the 12 HPAI-infected flocks, 11, 8, 8, 7 were detected for thresholds of 7.4, 8.6, 10.8, 14.1 times the moving-average, respectively. Detection with the moving-average happened between one day before and two days after the day of detection with the one-day 0.25% threshold:

```r
summary(t7, digits = 2)
```

```
##       7.4              8.6              10.8             14.1      
##  Min.   :-1.00   Min.   :-1.00   Min.   :-1.00   Min.   :-1.00  
##  1st Qu.:-1.00   1st Qu.:-1.00   1st Qu.:-1.00   1st Qu.:-1.00  
##  Median :-1.00   Median :-1.00   Median :-1.00   Median :-1.00  
##  Mean   :-0.18   Mean   :-0.38   Mean   :-0.38   Mean   :-0.29  
##  3rd Qu.: 0.50   3rd Qu.: 0.00   3rd Qu.: 0.00   3rd Qu.: 0.00  
##  Max.   : 2.00   Max.   : 2.00   Max.   : 2.00   Max.   : 2.00  
##  NA's   :1       NA's   :4       NA's   :4       NA's   :5      
```

```r
sp7 <-
  length(levels(dat_healthy$id)) -
  sapply(thrs3, function(i) {
    sum(sapply(levels(dat_healthy$id), function(j) {
      any(
        dat_healthy$rollmean_trigger[dat_healthy$id == j] >= i &
          c(FALSE, dat_healthy$rollmean_trigger[dat_healthy$id == j][
            -sum(dat_healthy$id == j)
          ] >= i),
        na.rm = TRUE
```

```r
      )
    }))
  })

fa7 <-
  sapply(thrs3, function(i) {
    sapply(levels(dat_healthy$id), function(j) {
      100 * sum(
        dat_healthy$rollmean_trigger[dat_healthy$id == j] >= i &
          c(FALSE, dat_healthy$rollmean_trigger[dat_healthy$id == j][
            -sum(dat_healthy$id == j)
          ] >= i),
        na.rm = TRUE
      ) /
        length(na.omit(dat_healthy$rollmean_trigger[dat_healthy$id == j]))
    })
  })
```

Among the 18 healthy flocks, 18, 18, 18, 18 had no alarm for thresholds of 7.4, 8.6, 10.8, 14.1 times the moving-average, respectively.
